# Interest of ^18^F-Fluorodeoxyglucose Positron Emission Tomography/Computed Tomography for Fever and Inflammatory Syndrome of Unknown Origin in Elderly Patients: A Retrospective Real-Life Single-Center Study from a University Referral Hospital

**DOI:** 10.3390/jcm14041188

**Published:** 2025-02-11

**Authors:** Carole Greuez, Noel Lorenzo-Villalba, Darejan Mamulashvili Bessac, Thomas Vogel, Cyrille Blondet, Jean-Christophe Weber, Georges Kaltenbach, Alessio Imperiale, Emmanuel Andrès

**Affiliations:** 1 Internal Medicine, Strasbourg University Hospital, 67000 Strasbourg, France; 2 Nuclear Medicine, Strasbourg University Hospital, 67000 Strasbourg, Francea.imperiale@icans.eu (A.I.); 3 Geriatric Department, Strasbourg University Hospital, 67000 Strasbourg, Francegeorges.kaltenbach@chru-strasbourg.fr (G.K.)

**Keywords:** elderly, ^18^F-FDG, PET, fever of unknown origin, inflammatory syndrome, infection

## Abstract

**Background:** Fever and inflammatory syndrome of unknown origin pose diagnostic challenges, particularly in elderly patients with atypical presentations. ^18^F-Fluorodeoxyglucose Positron Emission Tomography/Computed Tomography (^18^F-FDG PET/CT) has proven useful in these cases, yet its role in geriatric populations remains underexplored. This study evaluates the impact of ^18^F-FDG PET/CT on the management of these conditions in elderly patients. **Methods:** A retrospective study of patients aged ≥75 years who underwent ^18^F-FDG PET/CT between 2013 and 2018 for unexplained fever or inflammatory syndrome was conducted. The primary outcome was the impact of ^18^F-FDG PET/CT on treatment decisions, defined as any change in treatment within 6 months of the scan request. Therapeutic changes included the initiation of new treatments or discontinuation of existing ones, regardless of the diagnosis. **Results:** Ninety-three patients (mean age: 82.2 years) were included. ^18^F-FDG PET/CT contributed to a definitive diagnosis in 30.8% of cases, with infections (19.8%), inflammatory diseases (19.8%), and malignancies (14.3%) being the most frequent diagnoses. Of the 61 patients who underwent further testing, 33 (39.3%) had targeted tests based on the ^18^F-FDG PET/CT findings. Histology was obtained for 28 patients, with 18 targeted biopsies. Therapeutic modifications occurred in 38.8% of cases, with new treatments initiated in 33.3% and treatment discontinued in 10%. False positives occurred in 15.2% of cases. **Conclusions:** ^18^F-FDG PET/CT is a valuable tool in managing elderly patients with unexplained fever or inflammatory syndrome, aiding diagnosis and therapeutic decisions. Its use should be considered in the elderly population but must be carefully weighed against the patient’s frailty and the available treatment options.

## 1. Introduction

Fever (FUO) and inflammation of unknown origin (IUO) are complex clinical conditions that pose significant diagnostic challenges. FUO is defined as a fever ≥38.3 °C lasting at least three weeks without a clear diagnosis despite extensive investigations, including appropriate inpatient evaluation or multiple outpatient consultations [1,2]. On the other hand, IUO has been more recently described as prolonged and perplexing inflammation with temperatures < 38.3 °C without an underlying diagnosis [3]. Over 200 potential causes have been identified, mainly infections, malignancies, and non-infectious inflammatory diseases (NIIDs) [4]. In Europe, approximately 30% of cases remain undiagnosed, with NIIDs accounting for 25% and infections for about 20% [5,6,7,8].

In elderly patients, age-related physiological changes may mask typical signs of infection or inflammation, such as fever and pain. Multiple comorbidities, immunosenescence, and frailty further complicate the diagnostic process, making it difficult to isolate a single cause [1,9]. Accordingly, the duration criteria of more than 3 weeks typically used for fever and inflammatory syndrome of unknown origin in younger patients may not be readily adapted to elderly adults. This clinical complexity often necessitates advanced diagnostic tools. In this clinical scenario, ^18^F-Fluorodeoxyglucose Positron Emission Tomography/Computed Tomography (^18^F-FDG PET/CT) has proven to be a valuable tool for detecting metabolic activity associated with infections, inflammation, and malignancies, particularly when conventional imaging techniques are inconclusive [10,11,12,13,14]. By identifying areas of increased metabolic activity early, ^18^F-FDG PET/CT could offer a powerful diagnostic approach for elderly patients with fever and inflammation of unknown origin [15,16,17,18].

Despite its potential, the specific role of ^18^F-FDG PET/CT in managing these conditions in geriatric populations remains largely underexplored. To the best of our knowledge, no other study has evaluated the therapeutic impact of ^18^F-FDG PET/CT in elderly patients. Dedicated studies are lacking, although this minimally invasive test could rapidly guide diagnosis and treatment tailored to the patient’s overall condition. Therefore, the present study aims to evaluate the impact of ^18^F-FDG PET/CT on the management of fever or inflammatory syndrome of unknown origin, with a focus on elderly patients in a real-life observational setting.

## 2. Materials and Methods

### 2.1. Population

This retrospective, monocentric study included patients referred to our institution for ^18^F-FDG PET/CT between May 2013 and December 2018. Eligibility criteria were age 75 or older, fever or inflammatory syndrome (CRP ≥ 30 mg/L according to Vanderschueren’s definition for FUO) [8], and no definitive diagnosis after initial clinical investigations, including microbiological tests, imaging, and serological assessments. Patients with an established diagnosis, recent malignancy (unless recurrence was suspected), or those unable to provide informed consent were excluded. Clinical, imaging, and biological data were retrieved from hospital databases, including demographic information, comorbidities, clinical features (fever, CRP levels, and other inflammatory markers), medical history, and therapeutic modifications. Date of examination request and date of ^18^F-FDG PET/CT performed were also collected. These modifications, including the initiation or cessation of treatments like antibiotics, corticosteroids, immunosuppressants, and chemotherapy, were analyzed in relation to the ^18^F-FDG PET/CT findings.

All patients provided written informed consent for the use of anonymized data, and this study was approved by the local Institutional Review Board on 12 July 2019 (Ethic code: FC/dossier 2019-46) and by *CNIL* (National Commission on Informatics and Liberties) according to French law on 4 October 2018 (declaration number: 2208067 v 0).

### 2.2. ^18^F-FDG PET/CT

^18^F-FDG PET/CT was performed approximately 60 min after the intravenous injection of 4.5 MBq/kg of ^18^F-FDG. A non-enhanced CT scan from the head to mid-thighs was acquired during normal breathing, followed by PET acquisition. PET data were reconstructed with CT-based attenuation correction using an iterative algorithm (OSEM) and displayed on a dedicated workstation. A nuclear medicine physician, aware of the clinical context, visually interpreted the scans as positive or negative, assessing hypermetabolic activity indicative of infection, inflammation, or malignancy. A positive result was defined as focal, pathologically increased uptake compared to surrounding tissue.

The ^18^F-FDG PET/CT findings were compared to histology (when available) or clinical follow-up, including physical examination, biological tests, conventional imaging, and microbiological investigations. The ^18^F-FDG PET/CT results were categorized as contributory (leading to a final diagnosis), non-contributory, or false positive. Patients were followed for at least six months to assess the impact of ^18^F-FDG PET/CT on therapeutic decisions and clinical outcomes.

### 2.3. Primary Outcome

The primary outcome was the impact of ^18^F-FDG PET/CT on treatment decisions, defined as any change in treatment within 6 months of the scan request. The primary endpoint was determined strictly independently of the ^18^F-FDG PET/CT scan result, whether or not it contributed to the final diagnosis. We conducted a detailed retrospective analysis of treatment histories, prescriptions, and hospitalization or consultation reports. Therapeutic changes included the initiation of new treatments or discontinuation of existing ones, regardless of the diagnosis. These treatments could be medical (e.g., antibiotics, corticosteroids, immunosuppressants), surgical, or radiotherapeutic, specifically targeting fever or inflammatory syndrome. In cases of palliative care, treatment discontinuation was also recorded as a therapeutic change.

### 2.4. Statistical Analysis

Descriptive statistics were used to summarize patient demographics, clinical features, and outcomes. Continuous variables were presented as mean ± standard deviation or median with interquartile range (IQR), as appropriate. Categorical variables were expressed as percentages. To compare continuous data, Student’s *t*-test or Wilcoxon’s test were applied. The Chi-squared or Fisher’s test was used for categorical variables. Bivariate analysis identified significant associations between patient characteristics and the likelihood of therapeutic changes following ^18^F-FDG PET/CT. Logistic regression assessed factors associated with positive diagnostic contributions and therapeutic modifications. Statistical significance was set at *p* < 0.05. Statistical analysis was performed with SPSS software (Statistical Package for the Social Sciences, IBM Corp. IBM SPSS Statistics for Windows, Version 29.0, Armonk, NY, USA: IBM Corp).

## 3. Results

### 3.1. Population

**Figure 1:**
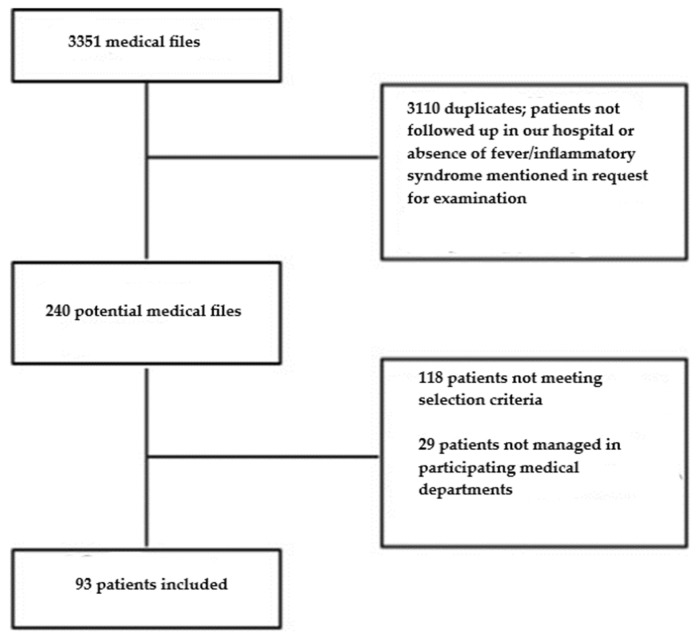
Flowchart.

A total of 93 patients were included (Figure 1).

In the sample, 52 of the individuals were men (55.9%) and the mean age was 82.2 years. Eighty-five (92.4%) cases were hospitalized in the internal medicine, geriatrics, or infectious diseases departments. The average time to obtain ^18^F-FDG PET/CT was 22 days (range: 1–120 days).

In total, 69 (74.2%) patients had hypertension, 62 (66.7%) had heart failure, 33 (36.3%) had renal insufficiency, and 24 (25.8%) had diabetes. Eleven patients (11.8%) were followed for neoplastic disease (Hodgkin’s lymphoma, mantle cell lymphoma, chronic lymphocytic leukemia, myeloproliferative syndrome, ovarian or bladder carcinoma, melanoma, and squamous cell carcinoma of the head and neck). There were 19 patients (20.7%) with a history of immunosuppression.

The average Charlson comorbidity score was 6.4, indicating a high number of comorbidities in these patients and a 10-year survival probability ranging from 0% to 2% on average. Seventy-three (80.2%) patients received at least five different medications/day at the time of ^18^F-FDG PET/CT. A total of 89 (96.7%) patients had biological inflammatory syndrome, and 28 patients met the criteria for prolonged inflammatory syndrome. The CRP mean value at the time of the ^18^F-FDG PET/CT scan was 107 mg/L, ranging from 19 to 300 mg/L. In total, 33 (35.9%) patients had fever and 11 met the criteria for fever of unknown origin (FUO). Twenty-nine patients (31.2%) had both fever and biological inflammatory syndrome.

No final diagnosis was identified in 38 (41.8%) patients, 18 (18.9%) patients had an infectious disease, and 18 (18.9%) patients had an NIID. Neoplastic causes were found in 13 (14.3%) patients and other etiologies in 4 (4.4%) additional patients. At 6 months, 16 deaths were reported (data missing for 17 patients) related to a final diagnosis of cancer in six patients, infection in four patients, and NIIDs in three patients. No diagnosis could be identified for the remaining three patients.

### 3.2. ^18^F-FDG PET/CT and Targeted Complementary Investigations

Twenty-eight patients (30.8%) showed ^18^F-FDG uptake abnormalities contributing to a definitive diagnosis, with infections (19.8%), inflammatory diseases (19.8%), and malignancies (14.3%) being the most frequent diagnoses.

After ^18^F-FDG PET/CT, 61 patients (72.6%; nine missing data) underwent additional complementary tests, with 33 patients (39.3%) receiving targeted tests based on PET/CT abnormalities. Histology was obtained for 28 patients (35%; 13 missing data), and in 18 cases, biopsies were targeted based on the PET/CT findings. Colonic focal ^18^F-FDG uptake led to colonoscopies with biopsies in five cases, revealing no abnormalities. Joint or vertebral uptake resulted in aspirations or biopsies for four patients. One patient, ultimately diagnosed with mesothelioma and managed palliatively, underwent a transesophageal echocardiogram for mitral valve uptake, ruling out endocarditis. Histology is not available in fragile patients for whom the decision has been made not to perform a more invasive examination. The performance of complementary tests, whether targeted or not, was not associated with therapeutic modification (*p* = 0.18) (Table 1).

### 3.3. Primary Outcome

Six months following the ^18^F-FDG PET/CT scan, therapeutic modification was observed in 31 patients (38.8%), with 4 patients experiencing both the initiation and cessation of treatment.

New treatments were introduced in 27 patients (33.3%). These included anti-infective therapies, corticosteroids, immunosuppressive treatments, colchicine, surgery, chemotherapy, and radiotherapy. The descriptive analysis separating the two components of the primary endpoint showed that among the 27 patients for whom a new treatment was introduced, 6 patients (7.4%, omitting missing data) were receiving anti-infective treatment, 4 patients (4.9%) were receiving corticosteroid therapy, 4 patients (4.9%) were receiving other immunosuppressive therapy, 2 patients (2.5%) were being treated with colchicine for microcrystalline arthritis, 2 patients (2.5%) were treated surgically, 3 patients (3.7%) were starting chemotherapy, 2 patients (2.5%) were treated exclusively with radiotherapy, and 4 patients (4.9%) had received several types of treatment (2 patients were treated by surgery and antibiotic therapy, respectively, for an infection on a total hip prosthesis and for endocarditis on a pacemaker lead, 1 patient was treated by vertebroplasty and anastrozole (Arimidex^®^) for breast cancer with bone metastases, and 1 patient was treated by corticosteroid therapy combined with hydroxyurea (Hydréa^®^) for chronic myelomonocytic leukemia).

**Figure 2:**
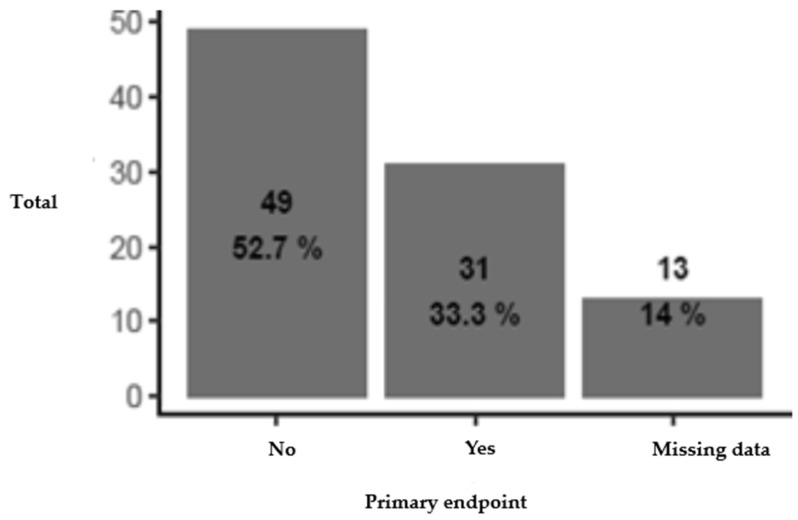
Distribution by primary endpoint (therapeutic modification). Note: percentages shown vary from Table 2 due to the inclusion of missing data in this presentation. Legend: No: number (%) patients not reaching the endpoint, Yes: No: number (%) patients reaching the endpoint. Four patients were concerned by both the introduction and discontinuation of treatment. For 12 patients, we did not have any information regarding the follow-up at our hospital after the PET, which made it impossible to conclude whether treatment had been introduced or discontinued within 6 months. Antibiotic therapy was initiated in one patient (followed-up for CLL), presenting an inflammatory syndrome in the setting of pachypleuritis in the left hemithorax, but we were unable to determine whether the discontinuation of antibiotic therapy was due to the PET result.

Treatment discontinuation occurred in eight patients (10%). In four cases, curative treatments were stopped as part of palliative care: two patients with mesothelioma and metastatic bladder carcinoma, one patient with critical limb ischemia who refused treatment, and one patient without a final diagnosis. In two additional cases, antibiotics were discontinued after confirming microcrystalline arthritis. Rituximab was stopped in one patient with rheumatoid arthritis and ganglionic tuberculosis and in another with follicular lymphoma and myositis (Table 2 and Figure 2).

### 3.4. Factors Associated with Therapeutic Modification

The presence of abnormality(ies) on ^18^F-FDG PET/CT related to the selected diagnosis was significantly associated with a therapeutic change. This association was not confirmed by multivariate analysis. In fact, there is a clear relationship between not having treatment and not having a diagnosis. This is a confounding factor, neutralized by adjustment in the multivariate analysis. For patients without an identified diagnosis, having an ^18^F-FDG PET scan or an ^18^F-FDG PET-CT scan without any particularities seems to be a good prognostic factor associated with more spontaneous remission.

Several factors were significantly associated with therapeutic modification. The time to perform ^18^F-FDG PET/CT was shorter in the group with therapeutic modification (median 6 vs. 22 days, *p* = 0.048). Fever was also significantly associated (16 patients (51.6%) with modification vs. 12 patients (24.5%) without, *p* = 0.025), as well as the FUO criteria (*p* = 0.049). Abnormal findings on ^18^F-FDG PET/CT related to the final diagnosis were strongly linked to therapeutic changes (17 patients (54.8%) with modification vs. 9 (18.4%) without, *p* = 0.002). The type of diagnosis was also significant (*p* = 0.001), and mortality at 6 months was higher in the therapeutic modification group (12 deaths (48.4%) vs. 4 deaths (10%), *p* = 0.004) (Table 3).

Concerning the multivariate analysis, the initial model included all variables with *p* < 0.2, as well as the level of dependence and albumin levels. Since the level of dependence had 16 missing data points, a second model was constructed without this variable. Then, the variables were excluded based on the AIC (Akaike Information Criterion) and Wald test (stepwise selection) until the maximum number of variables allowed by the number of events (3) was reached. All interactions were tested. The Hosmer and Lemeshow test did not show poor model fit. The sensitivity analysis, after removing the most influential observations based on Cook’s distances, did not alter the results. Fever was the only significant variable in both models (OR = 3.77, 95% CI [1.39–10.77], *p* = 0.001; OR = 3.34, 95% CI [1.14–10.3], *p* = 0.03). The other ORs are provided for reference but are not significantly different from one and are not well estimated, as indicated by their confidence intervals. The FUO criteria were excluded due to correlation with fever. A bivariate analysis comparing patients with and without fever showed significant differences in treatment initiation (15 (53.6%) with fever vs. 12 (22.6%) without, *p* = 0.01) and therapeutic modification (16 (57.1%) with fever vs. 15 (28.8%) without, *p* = 0.025) (Table 4).

### 3.5. Absence of Therapeutic Modification

For patients without therapeutic modification, several management strategies were observed. Treatment initially introduced for unexplained fever or biological inflammatory syndrome was either continued or simple monitoring or therapeutic abstention was chosen. Data were missing for 10 patients. Among the 25 patients (30.9%) treated before ^18^F-FDG PET/CT, treatment remained unchanged, with half receiving corticosteroids. Simple monitoring was chosen for 20 patients (24.6%), while 7 patients (8.9%) achieved complete symptom remission without specific treatment before the scan.

## 4. Discussion

To our knowledge, this study is unique in assessing not only the diagnostic efficiency of ^18^F-FDG PET/CT in elderly patients with fever or inflammatory syndrome of unknown origin but also its impact on subsequent therapeutic management. This is particularly relevant for the geriatric population, which is projected to grow significantly in the coming decades. Our findings highlight the role of ^18^F-FDG PET/CT in real-world clinical practice, where therapeutic changes occurred in 38.8% of patients, despite a 40% undiagnosed rate. While it is difficult to quantify the exact contribution of ^18^F-FDG PET/CT to these changes, it is evident that it played a role in clinical decision-making. The early use of ^18^F-FDG PET/CT, as suggested by our results, can help guide further testing and potentially reduce unnecessary procedures, thereby improving cost-effectiveness.

Our cohort included a significant number of cases with no identified diagnosis and a comparable distribution of infectious and non-infectious inflammatory diseases (NIIDs). The increased susceptibility to infections in elderly patients is likely influenced by immunosenescence and the presence of multimorbidity. Conversely, the high incidence of NIIDs observed could be attributed to the broad inclusion criteria of this study, which aimed to reflect real-life clinical scenarios. These criteria included no time limits for fever or inflammatory syndrome duration, as well as the inclusion of immunocompromised patients. The use of CRP ≥ 30 mg/L as a threshold aligns with Vanderschueren’s criteria (3), although this value can vary across studies, ranging from 7 to 50 mg/L. A relevant study by Bleekers-Rovers et al. [6] examined a cohort of 48 patients with suspected infection or inflammation but who did not meet the criteria for FUO or prolonged inflammatory syndrome. The average age of the participants in their study was 61 years. In their cohort, 58% of cases were attributed to infections, 7% to NIIDs, 5% to neoplastic causes, and 5% to other diagnoses, with 18% remaining undiagnosed. In comparison, their study found a higher proportion of infectious diseases and fewer undiagnosed cases than our cohort. This difference may be explained by their inclusion of patients with positive blood cultures, which likely skewed the results toward infectious causes.

The role of ^18^F-FDG PET/CT in immunocompromised patients is invaluable for identifying infectious, inflammatory, and neoplastic causes of fever or inflammatory syndrome of unknown origin [19,20]. However, in elderly immunocompromised patients, specific data in the literature remain sparse. Interestingly, in our cohort, we observed prevalent inflammatory causes in this group. This can be explained by the inclusion of patients in flare-ups of their inflammatory conditions, where ^18^F-FDG PET/CT was requested to rule out other potential causes. Despite conditions such as rheumatoid arthritis or polymyalgia rheumatica, no active disease was detected in these patients during clinical examination. Additionally, two patients were diagnosed with microcrystalline arthritis (gout or chondrocalcinosis) and treated successfully with colchicine. Although the use of ^18^F-FDG PET/CT in these cases may seem excessive or premature, it reflects real-world clinical decision-making, demonstrating the complexity of patient selection based on the available information at the time of the scan request.

This study’s focus on subsequent management is novel. While many studies have assessed the diagnostic efficiency of ^18^F-FDG PET/CT in cases of FUO or prolonged inflammatory syndrome [10,11,12,13,14,15,16,17,18], few have addressed its impact on management decisions. This issue is particularly important in the geriatric population, where clinical decision-making is often more complex due to the presence of multimorbidity and frailty. Our real-world study provides valuable insight into clinical practices in a tertiary care university hospital, offering data that can inform quality improvement efforts in healthcare. Notably, a therapeutic modification occurred in 38.8% of patients, despite the 40% undiagnosed rate. Although the direct contribution of ^18^F-FDG PET/CT to these therapeutic changes cannot be fully determined, it is clear that the results from the exam, whether positive or negative, played an important role in guiding treatment decisions.

During the five-year study period, 93 patients were included, representing less than 3% of the 3351 patients aged 75 and older who underwent ^18^F-FDG PET/CT in the same period. Although the cases in our cohort were clinically important for the physicians involved, they represent a relatively rare occurrence. The early use of ^18^F-FDG PET/CT as a comprehensive approach in the diagnostic work-up of unexplained fevers and inflammatory syndromes has been highlighted as a means to reduce the number of additional tests required. This reduction could ultimately lead to improved cost-effectiveness from a medico-economic perspective. However, our real-world data show that a substantial number of additional tests are still prescribed both before and after ^18^F-FDG PET/CT. Consequently, it remains uncertain whether healthcare costs are actually reduced.

This work also emphasizes the importance of avoiding premature testing. While ^18^F-FDG PET/CT can be a valuable diagnostic tool, it is important to first revisit the patient’s medical history and conduct a thorough clinical examination to identify any overlooked clues. In cases where the symptoms are mild and there are no severe signs or poor tolerance, a more cautious approach can be taken, allowing time for reflection and potentially delaying the therapeutic approach. For example, in cases of falls, mild inflammatory syndromes often resolve spontaneously within a few days without specific treatment [21]. If the condition persists, its progression can provide additional clues, leading to a diagnosis. In these cases, the utility of ^18^F-FDG PET/CT is limited, as the likelihood of detecting metabolic abnormalities is low, and no specific treatment would be necessary. By carefully selecting patients, the chances of obtaining a valuable result from ^18^F-FDG PET/CT, which will guide subsequent management, are significantly increased.

It is well recognized that ^18^F-FDG PET/CT false positives can occur, particularly in elderly patients, due to various physiological factors such as inflammation, infections, or age-related metabolic changes. These false positives can lead to unnecessary additional testing or treatments, which may have unintended consequences, including increased risk of morbidity or unnecessary interventions. To mitigate the risks associated with false positives in FDG PET/CT, especially in elderly patients, it is crucial to integrate clinical context with imaging results. By carefully correlating imaging findings with the patient’s medical history, comorbidities, and physical examination, clinicians can better interpret ambiguous results. In cases where uncertainty remains, follow-up imaging or biopsies may be necessary to confirm the diagnosis, preventing unnecessary treatments. A multidisciplinary approach involving various specialists ensures a comprehensive interpretation of the results, especially in complex cases. Moreover, educating healthcare providers on the limitations of ^18^F-FDG PET/CT will further enhance diagnostic accuracy, minimizing the risks of misinterpretation.

In approximately one-third of the patients, treatment had already been introduced before ^18^F-FDG PET/CT and continued without modification. Adding this to the 38.8% of patients who experienced a therapeutic modification, we can conclude that nearly 70% of the patients had a treatment decision made, whether modified or not.

As a retrospective study, our work is subject to inherent biases, including missing data and potential errors in the data collection. While we made efforts to minimize these issues, such as verifying treatment dates in the electronic medical records, a substantial amount of critical geriatric data were missing. Moreover, the descriptive nature of this study prevents us from establishing direct cause-and-effect relationships between the PET/CT results and therapeutic modifications.

Future studies should focus on optimizing the timing of ^18^F-FDG PET/CT, striking a balance between the need for early diagnosis and the risks of premature testing. One potential avenue is the assessment of frailty, which could help determine whether the test is justified in individual patients. This approach could improve diagnostic efficiency and patient outcomes, especially in the elderly population.

## 5. Conclusions

^18^F-FDG PET/CT should be considered in elderly patients with unexplained fever or inflammatory syndrome, particularly when a thorough clinical examination and initial tests fail to provide a diagnosis. The non-invasive nature and ability to target diagnostic procedures make ^18^F-FDG PET/CT a valuable tool in clinical practice, but its use must be carefully weighed against the patient’s frailty and the available treatment options. In such cases, it is crucial to understand what is expected from the examination and what can be offered to the patient based on the results obtained.

## Figures and Tables

**Table 1:** General characteristics of the population.

Variable	n = 93 (%)	Standard Deviation (SD)	Missing Data
Mean age (years)	82.18	5.05	0
Men	52 (55.9)		0
Mean weight kg	70.13	15.94	2
Mean body weight index kg/m^2^	25.55	4.99	4
Living at home	87 (94.6)		1
Dependent	47 (61)		16
Medical antecedents:			
- Hypertension	69 (74.2)		0
- Heart Failure	62 (66.7)		0
- Stroke	15 (16.1)		0
- Vascular peripheral disease	23 (24.7)		0
- Diabetes	24 (25.8)		0
- Chronic renal failure	33 (36.3)		2
- Neoplasia in remission	17 (18.3)		0
- Active neoplasia	11 (11.8)		0
- Immunosuppression	19 (20.7)		1
- Dementia	22 (41.5)		40
- Parkinson	3 (3.2)		0
- Falls	22 (44.9)		44
- Malnutrition	42 (51.2)		11
- Albumin (g/L)	33.98	5.85	11
- Smoking	1 (2)		43
- Alcohol consumption (>14 units a week)	5 (16.7)		63
Polymedication *	73 (80.2)		2
Mean Charlson score	6.42	2.30	0
Patient management:			
- Hospitalization (days)	85 (92.4)		1
- Mean hospital stay (days)	35.52	32.68	5
Clinical presentation:			
- Fever	33 (35.9)		1
- FUO criteria	11 (12.2)		3
- Inflammatory syndrome	89 (96.7)		1
- Prolonged Unexplained Biological Inflammatory Syndrome	28 (33.7)		10
- PCR mg/L	107	68.22	4
Mean time PET-CT (days)	22.4	20.1	0
PET/CT abnormality(ies) related to diagnosis	28 (30.8)		2
Final diagnosis:			
- No diagnosis	38 (41.8)		2
- Infection	18 (19.8)		2
- Non-infectious inflammatory disease	18 (19.8)		2
- Neoplasia	13 (14.3)		2
- Others	4 (4.4)		2
Death at 6 months	16 (21.1)		17

Legend: * taking several treatments, including at least five different medications; FUO: fever of unknown origin; PCR: protein C-reactive.

**Table 2:** Primary endpoint (therapeutic modifications).

Variables	n = 93 (%)	Missing Data
Introduction	27 (33.3)	12
Discontinuation	8 (10)	13
Primary endpoint:		
Introduction and discontinuation	31 (38.8)	13

**Table 3:** Bivariate analysis of factors associated with the primary outcome (therapeutic modification).

Variable.	Median	Q1	Q3	Median	Q1	Q3	*p*
	No Treatment Modification n = 49			Treatment Modification n = 31			
Age (years)	81	78	85	82	78	87.5	0.703
BMI (kg/m^2^)	24.46	21.48	29.37	25.00	20.92	28.14	0.775
Charlson’s score	6	5	7	7	5	8.5	0.562
Hospital stay (days)	22	10.5	44	30	15	68	0.174
^18^F-FDG PET/CT delay (days)	22	6	36	6	4.5	26	0.048
Albumin (g/L)	34	29	38.3	34.5	30	37.25	0.80
PCR (mg/L)	85	50	141.8	109	68	146.5	0.428

Legend: BMI: body weight index; PCR: protein C-reactive.

**Table 4:** Analysis of factors significantly associated with the primary outcome (therapeutic modification).

Model 1				
Variable	OR PE	CI < 95	CI > 95	*p*
Fever	3.77	1.39	10.77	0.01
Inflammatory disease	0.39	0.11	1.25	0.13
Hypertension	2.57	0.8	9.52	0.13
**Model 2**				
Variable	OR PE	CI < 95	CI > 95	*p*
Delay	0.97	0.94	1	0.08
Fever	3.34	1.14	10.3	0.03
Level of dependence	1.36	0.78	2.45	0.28

Legend: PE: primary endpoint; OR = odds ratio; CI 95 = 95% confidence interval significance if *p* < 0.05 and CI 95% excludes 1.

## Data Availability

The data presented in this study are available on request from the corresponding author. The data are not publicly available due to privacy restrictions.
